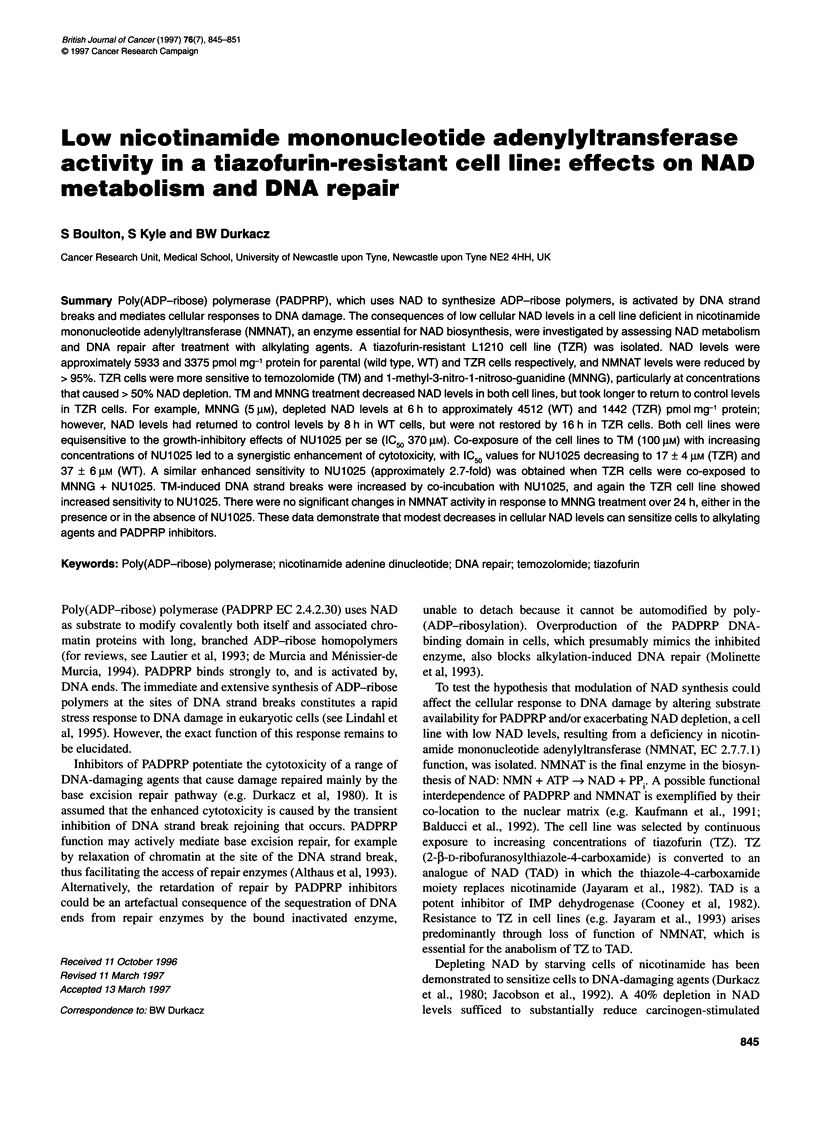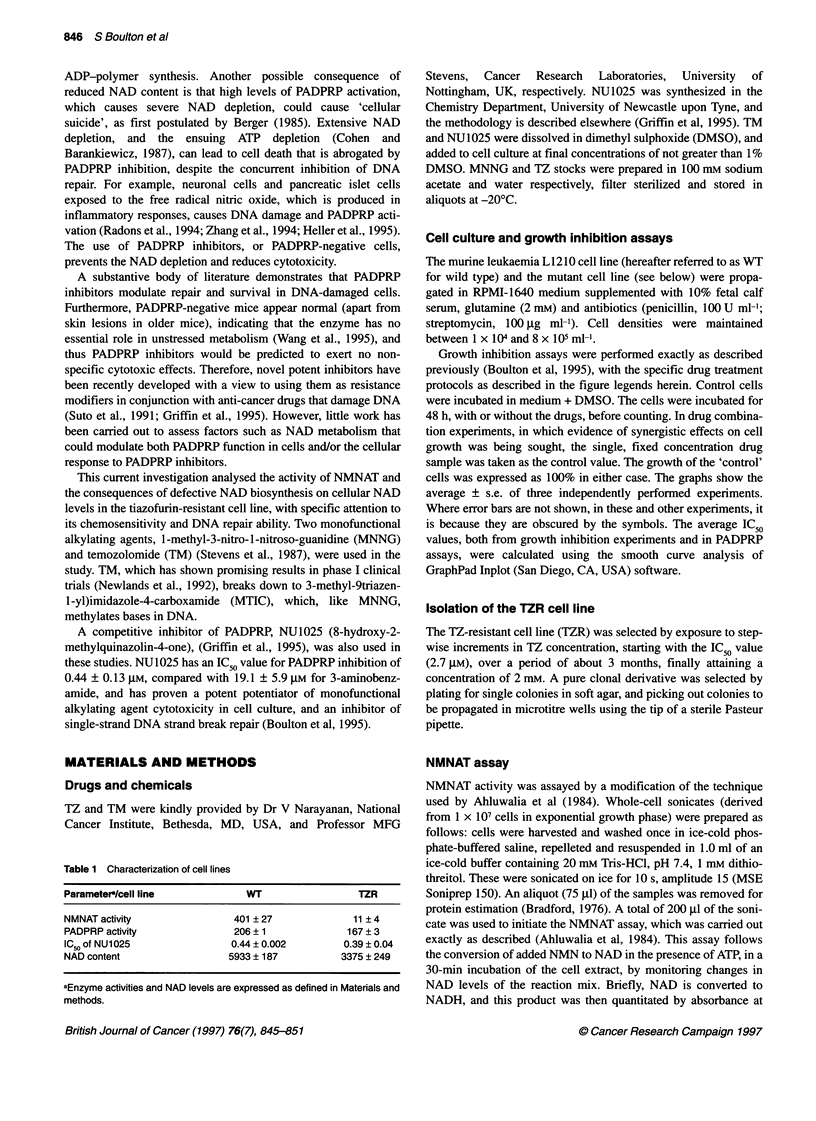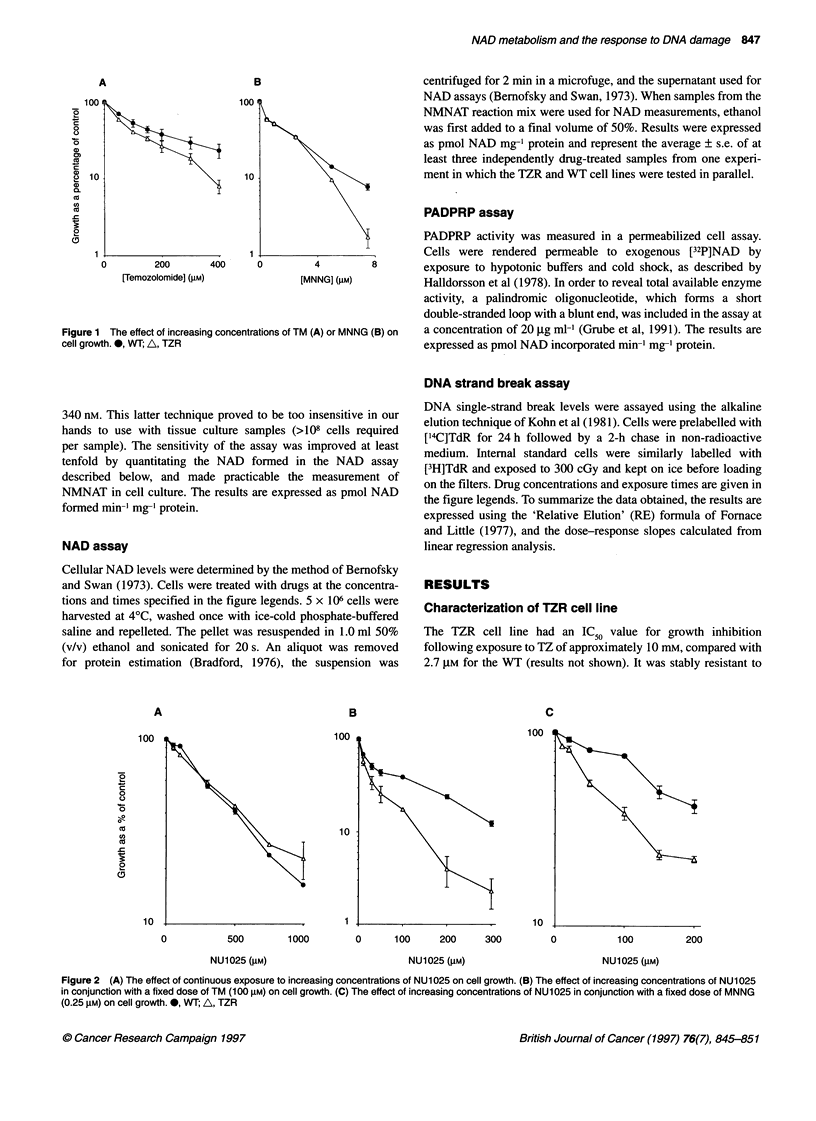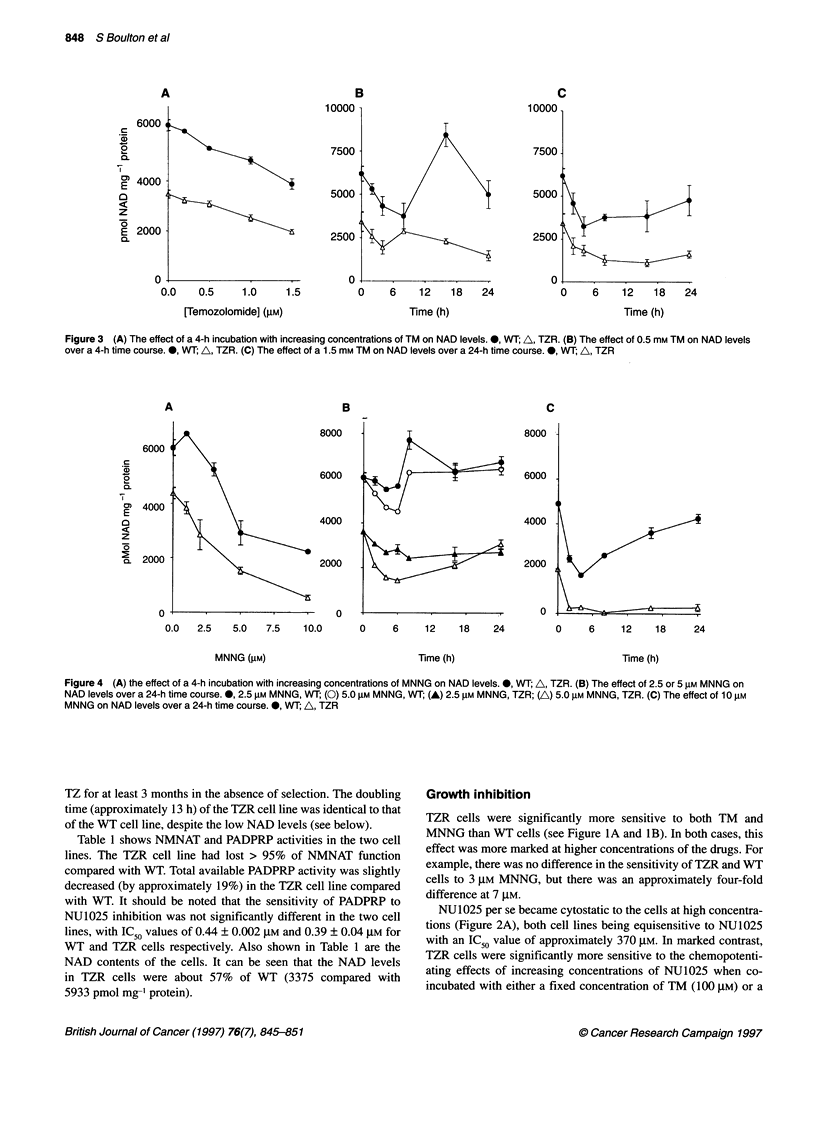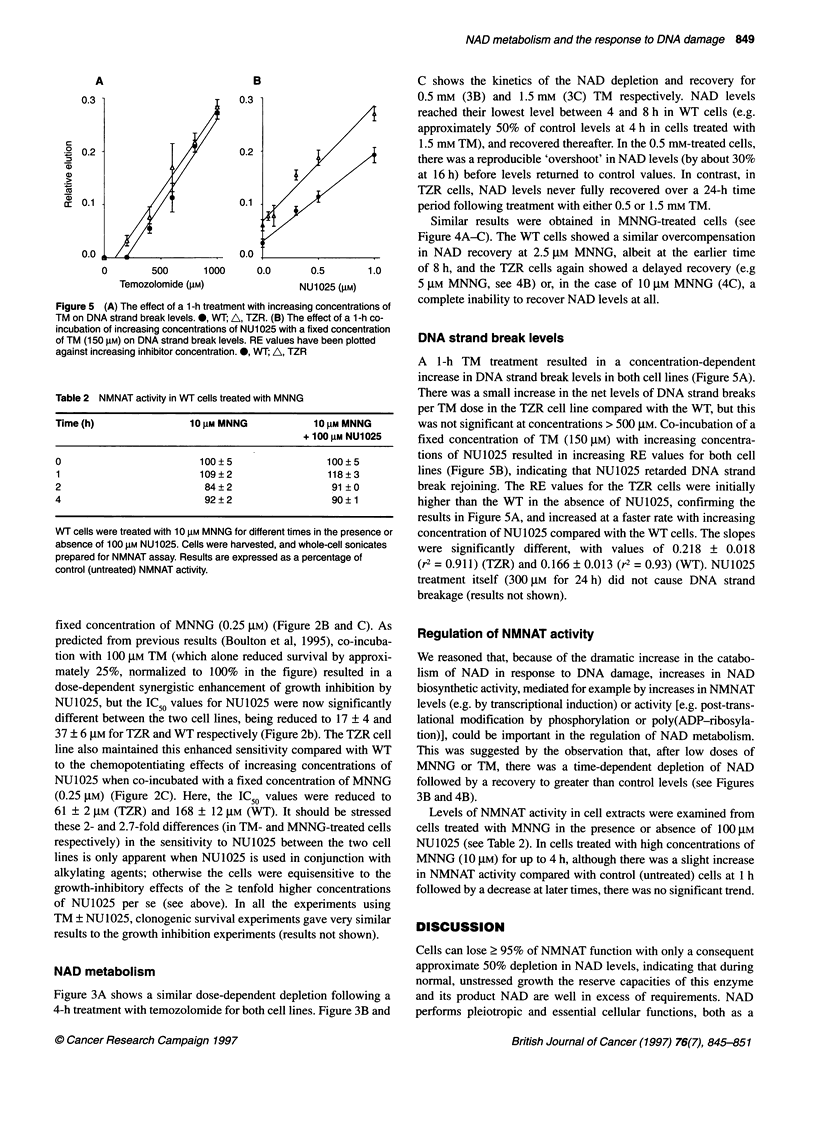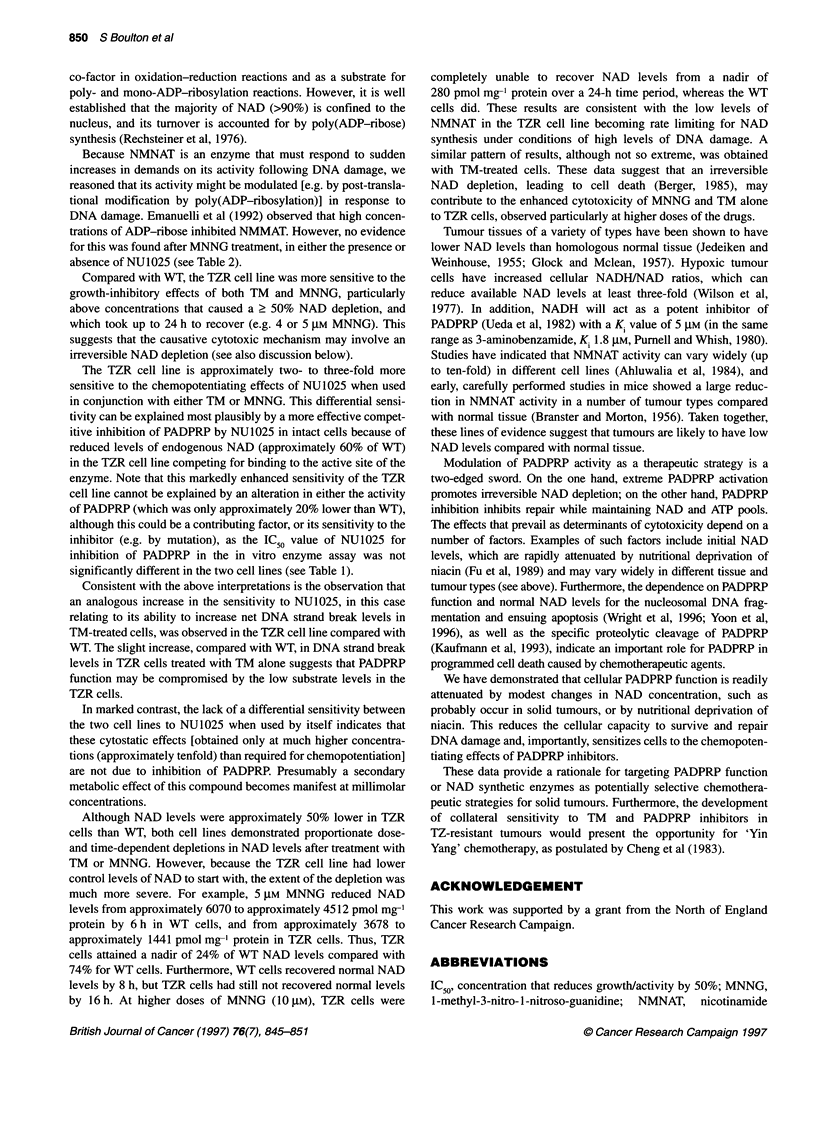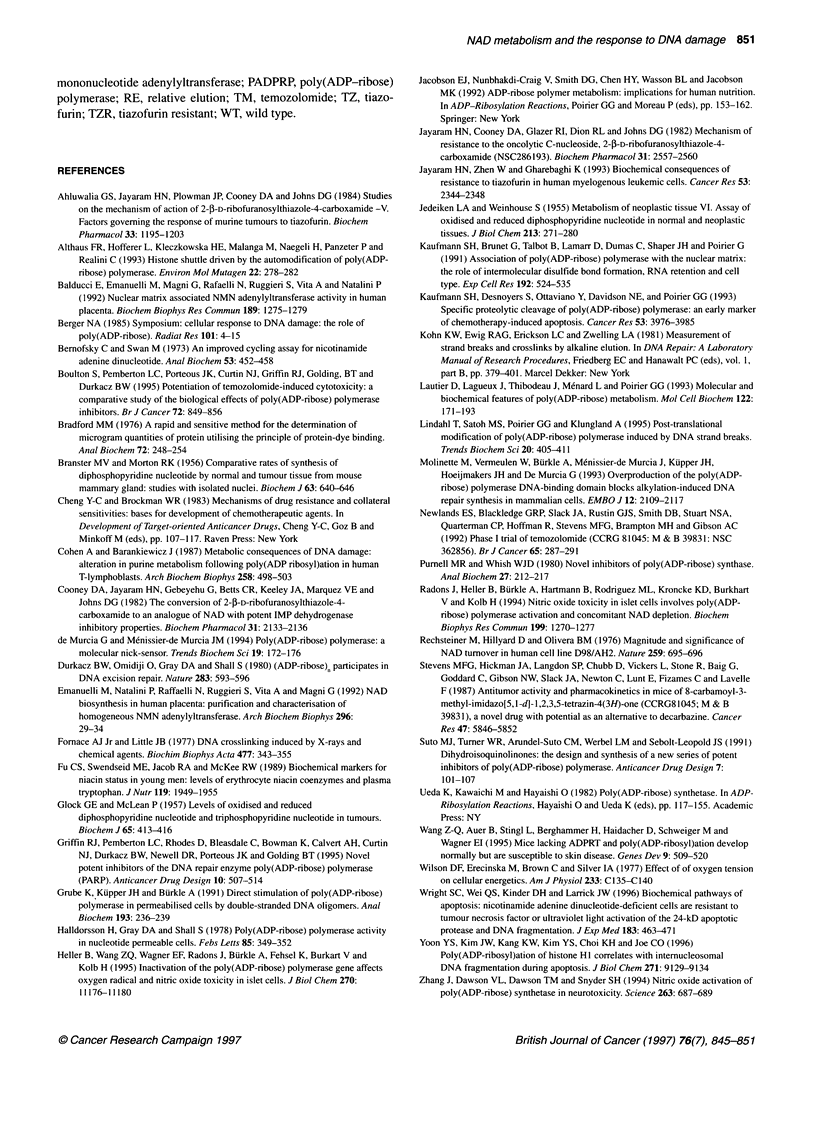# Low nicotinamide mononucleotide adenylyltransferase activity in a tiazofurin-resistant cell line: effects on NAD metabolism and DNA repair.

**DOI:** 10.1038/bjc.1997.473

**Published:** 1997

**Authors:** S. Boulton, S. Kyle, B. W. Durkacz

**Affiliations:** Cancer Research Unit, Medical School, University of Newcastle upon Tyne, UK.

## Abstract

Poly(ADP-ribose) polymerase (PADPRP), which uses NAD to synthesize ADP-ribose polymers, is activated by DNA strand breaks and mediates cellular responses to DNA damage. The consequences of low cellular NAD levels in a cell line deficient in nicotinamide mononucleotide adenylyltransferase (NMNAT), an enzyme essential for NAD biosynthesis, were investigated by assessing NAD metabolism and DNA repair after treatment with alkylating agents. A tiazofurin-resistant L1210 cell line (TZR) was isolated. NAD levels were approximately 5933 and 3375 pmol mg(-1) protein for parental (wild type, WT) and TZR cells respectively, and NMNAT levels were reduced by > 95%. TZR cells were more sensitive to temozolomide (TM) and 1-methyl-3-nitro-1-nitroso-guanidine (MNNG), particularly at concentrations that caused > 50% NAD depletion. TM and MNNG treatment decreased NAD levels in both cell lines, but took longer to return to control levels in TZR cells. For example, MNNG (5 microM), depleted NAD levels at 6 h to approximately 4512 (WT) and 1442 (TZR) pmol mg(-1) protein; however, NAD levels had returned to control levels by 8 h in WT cells, but were not restored by 16 h in TZR cells. Both cell lines were equisensitive to the growth-inhibitory effects of NU1025 per se (IC50 370 microM). Co-exposure of the cell lines to TM (100 microM) with increasing concentrations of NU1025 led to a synergistic enhancement of cytotoxicity, with IC50 values for NU1025 decreasing to 17 +/- 4 microM (TZR) and 37 +/- 6 microM (WT). A similar enhanced sensitivity to NU1025 (approximately 2.7-fold) was obtained when TZR cells were co-exposed to MNNG + NU1025. TM-induced DNA strand breaks were increased by co-incubation with NU1025, and again the TZR cell line showed increased sensitivity to NU1025. There were no significant changes in NMNAT activity in response to MNNG treatment over 24 h, either in the presence or in the absence of NU1025. These data demonstrate that modest decreases in cellular NAD levels can sensitize cells to alkylating agents and PADPRP inhibitors.


British Journal of Cancer (1997) 76(7), 845–851
© 1997 Cancer Research Campaign

### Low nicotinamide mononucleotide adenylyltransferase activity in a tiazofurin-resistant cell line: effects on NAD metabolism and DNA repair

**S Boulton, S Kyle and BW Durkacz**

Cancer Research Unit, Medical School, University of Newcastle upon Tyne, Newcastle upon Tyne NE2 4HH, UK

**Summary** Poly(ADP–ribose) polymerase (PADPRP), which uses NAD to synthesize ADP–ribose polymers, is activated by DNA strand breaks and mediates cellular responses to DNA damage. The consequences of low cellular NAD levels in a cell line deficient in nicotinamide mononucleotide adenylyltransferase (NMNAT), an enzyme essential for NAD biosynthesis, were investigated by assessing NAD metabolism and DNA repair after treatment with alkylating agents. A tiazofurin-resistant L1210 cell line (TZR) was isolated. NAD levels were approximately 5933 and 3375 pmol $mg^{-1}$ protein for parental (wild type, WT) and TZR cells respectively, and NMNAT levels were reduced by > 95%. TZR cells were more sensitive to temozolomide (TM) and 1-methyl-3-nitro-1-nitroso-guanidine (MNNG), particularly at concentrations that caused > 50% NAD depletion. TM and MNNG treatment decreased NAD levels in both cell lines, but took longer to return to control levels in TZR cells. For example, MNNG (5 μM), depleted NAD levels at 6 h to approximately 4512 (WT) and 1442 (TZR) pmol $mg^{-1}$ protein; however, NAD levels had returned to control levels by 8 h in WT cells, but were not restored by 16 h in TZR cells. Both cell lines were equisensitive to the growth-inhibitory effects of NU1025 per se ($IC_{50}$ 370 μM). Co-exposure of the cell lines to TM (100 μM) with increasing concentrations of NU1025 led to a synergistic enhancement of cytotoxicity, with $IC_{50}$ values for NU1025 decreasing to 17 ± 4 μM (TZR) and 37 ± 6 μM (WT). A similar enhanced sensitivity to NU1025 (approximately 2.7-fold) was obtained when TZR cells were co-exposed to MNNG + NU1025. TM-induced DNA strand breaks were increased by co-incubation with NU1025, and again the TZR cell line showed increased sensitivity to NU1025. There were no significant changes in NMNAT activity in response to MNNG treatment over 24 h, either in the presence or in the absence of NU1025. These data demonstrate that modest decreases in cellular NAD levels can sensitize cells to alkylating agents and PADPRP inhibitors.

**Keywords:** Poly(ADP–ribose) polymerase; nicotinamide adenine dinucleotide; DNA repair; temozolomide; tiazofurin

Poly(ADP–ribose) polymerase (PADPRP EC 2.4.2.30) uses NAD as substrate to modify covalently both itself and associated chromatin proteins with long, branched ADP–ribose homopolymers (for reviews, see Lautier et al, 1993; de Murcia and Ménissier-de Murcia, 1994). PADPRP binds strongly to, and is activated by, DNA ends. The immediate and extensive synthesis of ADP–ribose polymers at the sites of DNA strand breaks constitutes a rapid stress response to DNA damage in eukaryotic cells (see Lindahl et al, 1995). However, the exact function of this response remains to be elucidated.

Inhibitors of PADPRP potentiate the cytotoxicity of a range of DNA-damaging agents that cause damage repaired mainly by the base excision repair pathway (e.g. Durkacz et al, 1980). It is assumed that the enhanced cytotoxicity is caused by the transient inhibition of DNA strand break rejoining that occurs. PADPRP function may actively mediate base excision repair, for example by relaxation of chromatin at the site of the DNA strand break, thus facilitating the access of repair enzymes (Althaus et al, 1993). Alternatively, the retardation of repair by PADPRP inhibitors could be an artefactual consequence of the sequestration of DNA ends from repair enzymes by the bound inactivated enzyme, unable to detach because it cannot be automodified by poly-(ADP–ribosylation). Overproduction of the PADPRP DNA-binding domain in cells, which presumably mimics the inhibited enzyme, also blocks alkylation-induced DNA repair (Molinette et al, 1993).

Received 11 October 1996
Revised 11 March 1997
Accepted 13 March 1997

Correspondence to: BW Durkacz

To test the hypothesis that modulation of NAD synthesis could affect the cellular response to DNA damage by altering substrate availability for PADPRP and/or exacerbating NAD depletion, a cell line with low NAD levels, resulting from a deficiency in nicotinamide mononucleotide adenylyltransferase (NMNAT, EC 2.7.7.1) function, was isolated. NMNAT is the final enzyme in the biosynthesis of NAD: NMN + ATP → NAD + $PP_i$. A possible functional interdependence of PADPRP and NMNAT is exemplified by their co-location to the nuclear matrix (e.g. Kaufmann et al., 1991; Balducci et al., 1992). The cell line was selected by continuous exposure to increasing concentrations of tiazofurin (TZ). TZ (2-β-D-ribofuranosylthiazole-4-carboxamide) is converted to an analogue of NAD (TAD) in which the thiazole-4-carboxamide moiety replaces nicotinamide (Jayaram et al., 1982). TAD is a potent inhibitor of IMP dehydrogenase (Cooney et al, 1982). Resistance to TZ in cell lines (e.g. Jayaram et al., 1993) arises predominantly through loss of function of NMNAT, which is essential for the anabolism of TZ to TAD.

Depleting NAD by starving cells of nicotinamide has been demonstrated to sensitize cells to DNA-damaging agents (Durkacz et al., 1980; Jacobson et al., 1992). A 40% depletion in NAD levels sufficed to substantially reduce carcinogen-stimulated

ADP–polymer synthesis. Another possible consequence of reduced NAD content is that high levels of PADPRP activation, which causes severe NAD depletion, could cause 'cellular suicide', as first postulated by Berger (1985). Extensive NAD depletion, and the ensuing ATP depletion (Cohen and Barankiewicz, 1987), can lead to cell death that is abrogated by PADPRP inhibition, despite the concurrent inhibition of DNA repair. For example, neuronal cells and pancreatic islet cells exposed to the free radical nitric oxide, which is produced in inflammatory responses, causes DNA damage and PADPRP activation (Radons et al., 1994; Zhang et al., 1994; Heller et al., 1995). The use of PADPRP inhibitors, or PADPRP-negative cells, prevents the NAD depletion and reduces cytotoxicity.

A substantive body of literature demonstrates that PADPRP inhibitors modulate repair and survival in DNA-damaged cells. Furthermore, PADPRP-negative mice appear normal (apart from skin lesions in older mice), indicating that the enzyme has no essential role in unstressed metabolism (Wang et al., 1995), and thus PADPRP inhibitors would be predicted to exert no non-specific cytotoxic effects. Therefore, novel potent inhibitors have been recently developed with a view to using them as resistance modifiers in conjunction with anti-cancer drugs that damage DNA (Suto et al., 1991; Griffin et al., 1995). However, little work has been carried out to assess factors such as NAD metabolism that could modulate both PADPRP function in cells and/or the cellular response to PADPRP inhibitors.

This current investigation analysed the activity of NMNAT and the consequences of defective NAD biosynthesis on cellular NAD levels in the tiazofurin-resistant cell line, with specific attention to its chemosensitivity and DNA repair ability. Two monofunctional alkylating agents, 1-methyl-3-nitro-1-nitroso-guanidine (MNNG) and temozolomide (TM) (Stevens et al., 1987), were used in the study. TM, which has shown promising results in phase I clinical trials (Newlands et al., 1992), breaks down to 3-methyl-9triazen-1-yl)imidazole-4-carboxamide (MTIC), which, like MNNG, methylates bases in DNA.

A competitive inhibitor of PADPRP, NU1025 (8-hydroxy-2-methylquinazolin-4-one), (Griffin et al., 1995), was also used in these studies. NU1025 has an $IC_{50}$ value for PADPRP inhibition of 0.44 ± 0.13 μM, compared with 19.1 ± 5.9 μM for 3-aminobenzamide, and has proven a potent potentiator of monofunctional alkylating agent cytotoxicity in cell culture, and an inhibitor of single-strand DNA strand break repair (Boulton et al, 1995).

### MATERIALS AND METHODS

### Drugs and chemicals

TZ and TM were kindly provided by Dr V Narayanan, National Cancer Institute, Bethesda, MD, USA, and Professor MFG Stevens, Cancer Research Laboratories, University of Nottingham, UK, respectively. NU1025 was synthesized in the Chemistry Department, University of Newcastle upon Tyne, and the methodology is described elsewhere (Griffin et al, 1995). TM and NU1025 were dissolved in dimethyl sulphoxide (DMSO), and added to cell culture at final concentrations of not greater than 1% DMSO. MNNG and TZ stocks were prepared in 100 mM sodium acetate and water respectively, filter sterilized and stored in aliquots at –20°C.

**Table 1** Characterization of cell lines

| Parameter[a]/cell line | WT | TZR |
|---|---|---|
| NMNAT activity | 401 ± 27 | 11 ± 4 |
| PADPRP activity | 206 ± 1 | 167 ± 3 |
| $IC_{50}$ of NU1025 | 0.44 ± 0.002 | 0.39 ± 0.04 |
| NAD content | 5933 ± 187 | 3375 ± 249 |

[a]Enzyme activities and NAD levels are expressed as defined in Materials and methods.

### Cell culture and growth inhibition assays

The murine leukaemia L1210 cell line (hereafter referred to as WT for wild type) and the mutant cell line (see below) were propagated in RPMI-1640 medium supplemented with 10% fetal calf serum, glutamine (2 mM) and antibiotics (penicillin, 100 U $ml^{-1}$; streptomycin, 100 μg $ml^{-1}$). Cell densities were maintained between $1 \times 10^4$ and $8 \times 10^5$ $ml^{-1}$.

Growth inhibition assays were performed exactly as described previously (Boulton et al, 1995), with the specific drug treatment protocols as described in the figure legends herein. Control cells were incubated in medium + DMSO. The cells were incubated for 48 h, with or without the drugs, before counting. In drug combination experiments, in which evidence of synergistic effects on cell growth was being sought, the single, fixed concentration drug sample was taken as the control value. The growth of the 'control' cells was expressed as 100% in either case. The graphs show the average ± s.e. of three independently performed experiments. Where error bars are not shown, in these and other experiments, it is because they are obscured by the symbols. The average $IC_{50}$ values, both from growth inhibition experiments and in PADPRP assays, were calculated using the smooth curve analysis of GraphPad Inplot (San Diego, CA, USA) software.

### Isolation of the TZR cell line

The TZ-resistant cell line (TZR) was selected by exposure to stepwise increments in TZ concentration, starting with the $IC_{50}$ value (2.7 μM), over a period of about 3 months, finally attaining a concentration of 2 mM. A pure clonal derivative was selected by plating for single colonies in soft agar, and picking out colonies to be propagated in microtitre wells using the tip of a sterile Pasteur pipette.

### NMNAT assay

NMNAT activity was assayed by a modification of the technique used by Ahluwalia et al (1984). Whole-cell sonicates (derived from $1 \times 10^7$ cells in exponential growth phase) were prepared as follows: cells were harvested and washed once in ice-cold phosphate-buffered saline, repelleted and resuspended in 1.0 ml of an ice-cold buffer containing 20 mM Tris-HCl, pH 7.4, 1 mM dithiothreitol. These were sonicated on ice for 10 s, amplitude 15 (MSE Soniprep 150). An aliquot (75 μl) of the samples was removed for protein estimation (Bradford, 1976). A total of 200 μl of the sonicate was used to initiate the NMNAT assay, which was carried out exactly as described (Ahluwalia et al, 1984). This assay follows the conversion of added NMN to NAD in the presence of ATP, in a 30-min incubation of the cell extract, by monitoring changes in NAD levels of the reaction mix. Briefly, NAD is converted to NADH, and this product was then quantitated by absorbance at

© Cancer Research Campaign 1997

**Figure 1** The effect of increasing concentrations of TM (**A**) or MNNG (**B**) on cell growth. ●, WT; △, TZR

340 nM. This latter technique proved to be too insensitive in our hands to use with tissue culture samples (>$10^8$ cells required per sample). The sensitivity of the assay was improved at least tenfold by quantitating the NAD formed in the NAD assay described below, and made practicable the measurement of NMNAT in cell culture. The results are expressed as pmol NAD formed $min^{-1}$ $mg^{-1}$ protein.

### NAD assay

Cellular NAD levels were determined by the method of Bernofsky and Swan (1973). Cells were treated with drugs at the concentrations and times specified in the figure legends. 5 × $10^6$ cells were harvested at 4°C, washed once with ice-cold phosphate-buffered saline and repelleted. The pellet was resuspended in 1.0 ml 50% (v/v) ethanol and sonicated for 20 s. An aliquot was removed for protein estimation (Bradford, 1976), the suspension was centrifuged for 2 min in a microfuge, and the supernatant used for NAD assays (Bernofsky and Swan, 1973). When samples from the NMNAT reaction mix were used for NAD measurements, ethanol was first added to a final volume of 50%. Results were expressed as pmol NAD $mg^{-1}$ protein and represent the average ± s.e. of at least three independently drug-treated samples from one experiment in which the TZR and WT cell lines were tested in parallel.

### PADPRP assay

PADPRP activity was measured in a permeabilized cell assay. Cells were rendered permeable to exogenous [$^{32}$P]NAD by exposure to hypotonic buffers and cold shock, as described by Halldorsson et al (1978). In order to reveal total available enzyme activity, a palindromic oligonucleotide, which forms a short double-stranded loop with a blunt end, was included in the assay at a concentration of 20 μg $ml^{-1}$ (Grube et al, 1991). The results are expressed as pmol NAD incorporated $min^{-1}$ $mg^{-1}$ protein.

### DNA strand break assay

DNA single-strand break levels were assayed using the alkaline elution technique of Kohn et al (1981). Cells were prelabelled with [$^{14}$C]TdR for 24 h followed by a 2-h chase in non-radioactive medium. Internal standard cells were similarly labelled with [$^{3}$H]TdR and exposed to 300 cGy and kept on ice before loading on the filters. Drug concentrations and exposure times are given in the figure legends. To summarize the data obtained, the results are expressed using the 'Relative Elution' (RE) formula of Fornace and Little (1977), and the dose–response slopes calculated from linear regression analysis.

### RESULTS

### Characterization of TZR cell line

The TZR cell line had an $IC_{50}$ value for growth inhibition following exposure to TZ of approximately 10 mM, compared with 2.7 μM for the WT (results not shown). It was stably resistant to

**Figure 2** (**A**) The effect of continuous exposure to increasing concentrations of NU1025 on cell growth. (**B**) The effect of increasing concentrations of NU1025 in conjunction with a fixed dose of TM (100 μM) on cell growth. (**C**) The effect of increasing concentrations of NU1025 in conjunction with a fixed dose of MNNG (0.25 μM) on cell growth. ●, WT; △, TZR

**Figure 3** (**A**) The effect of a 4-h incubation with increasing concentrations of TM on NAD levels. ●, WT; △, TZR. (**B**) The effect of 0.5 mM TM on NAD levels over a 4-h time course. ●, WT; △, TZR. (**C**) The effect of a 1.5 mM TM on NAD levels over a 24-h time course. ●, WT; △, TZR

**Figure 4** (**A**) the effect of a 4-h incubation with increasing concentrations of MNNG on NAD levels. ●, WT; △, TZR. (**B**) The effect of 2.5 or 5 μM MNNG on NAD levels over a 24-h time course. ●, 2.5 μM MNNG, WT; (○) 5.0 μM MNNG, WT; (▲) 2.5 μM MNNG, TZR; (△) 5.0 μM MNNG, TZR. (**C**) The effect of 10 μM MNNG on NAD levels over a 24-h time course. ●, WT; △, TZR

TZ for at least 3 months in the absence of selection. The doubling time (approximately 13 h) of the TZR cell line was identical to that of the WT cell line, despite the low NAD levels (see below).

Table 1 shows NMNAT and PADPRP activities in the two cell lines. The TZR cell line had lost > 95% of NMNAT function compared with WT. Total available PADPRP activity was slightly decreased (by approximately 19%) in the TZR cell line compared with WT. It should be noted that the sensitivity of PADPRP to NU1025 inhibition was not significantly different in the two cell lines, with $IC_{50}$ values of 0.44 ± 0.002 μM and 0.39 ± 0.04 μM for WT and TZR cells respectively. Also shown in Table 1 are the NAD contents of the cells. It can be seen that the NAD levels in TZR cells were about 57% of WT (3375 compared with 5933 pmol $mg^{-1}$ protein).

### Growth inhibition

TZR cells were significantly more sensitive to both TM and MNNG than WT cells (see Figure 1A and 1B). In both cases, this effect was more marked at higher concentrations of the drugs. For example, there was no difference in the sensitivity of TZR and WT cells to 3 μM MNNG, but there was an approximately four-fold difference at 7 μM.

NU1025 per se became cytostatic to the cells at high concentrations (Figure 2A), both cell lines being equisensitive to NU1025 with an $IC_{50}$ value of approximately 370 μM. In marked contrast, TZR cells were significantly more sensitive to the chemopotentiating effects of increasing concentrations of NU1025 when co-incubated with either a fixed concentration of TM (100 μM) or a

© Cancer Research Campaign 1997

**Figure 5** (**A**) The effect of a 1-h treatment with increasing concentrations of TM on DNA strand break levels. ●, WT; △, TZR. (**B**) The effect of a 1-h co-incubation of increasing concentrations of NU1025 with a fixed concentration of TM (150 μM) on DNA strand break levels. RE values have been plotted against increasing inhibitor concentration. ●, WT; △, TZR

**Table 2** NMNAT activity in WT cells treated with MNNG

| Time (h) | 10 μM MNNG | 10 μM MNNG + 100 μM NU1025 |
|---|---|---|
| 0 | 100 ± 5 | 100 ± 5 |
| 1 | 109 ± 2 | 118 ± 3 |
| 2 | 84 ± 2 | 91 ± 0 |
| 4 | 92 ± 2 | 90 ± 1 |

WT cells were treated with 10 μM MNNG for different times in the presence or absence of 100 μM NU1025. Cells were harvested, and whole-cell sonicates prepared for NMNAT assay. Results are expressed as a percentage of control (untreated) NMNAT activity.

fixed concentration of MNNG (0.25 μM) (Figure 2B and C). As predicted from previous results (Boulton et al, 1995), co-incubation with 100 μM TM (which alone reduced survival by approximately 25%, normalized to 100% in the figure) resulted in a dose-dependent synergistic enhancement of growth inhibition by NU1025, but the $IC_{50}$ values for NU1025 were now significantly different between the two cell lines, being reduced to 17 ± 4 and 37 ± 6 μM for TZR and WT respectively (Figure 2b). The TZR cell line also maintained this enhanced sensitivity compared with WT to the chemopotentiating effects of increasing concentrations of NU1025 when co-incubated with a fixed concentration of MNNG (0.25 μM) (Figure 2C). Here, the $IC_{50}$ values were reduced to 61 ± 2 μM (TZR) and 168 ± 12 μM (WT). It should be stressed these 2- and 2.7-fold differences (in TM- and MNNG-treated cells respectively) in the sensitivity to NU1025 between the two cell lines is only apparent when NU1025 is used in conjunction with alkylating agents; otherwise the cells were equisensitive to the growth-inhibitory effects of the ≥ tenfold higher concentrations of NU1025 per se (see above). In all the experiments using TM ± NU1025, clonogenic survival experiments gave very similar results to the growth inhibition experiments (results not shown).

### NAD metabolism

Figure 3A shows a similar dose-dependent depletion following a 4-h treatment with temozolomide for both cell lines. Figure 3B and C shows the kinetics of the NAD depletion and recovery for 0.5 mM (3B) and 1.5 mM (3C) TM respectively. NAD levels reached their lowest level between 4 and 8 h in WT cells (e.g. approximately 50% of control levels at 4 h in cells treated with 1.5 mM TM), and recovered thereafter. In the 0.5 mM-treated cells, there was a reproducible 'overshoot' in NAD levels (by about 30% at 16 h) before levels returned to control values. In contrast, in TZR cells, NAD levels never fully recovered over a 24-h time period following treatment with either 0.5 or 1.5 mM TM.

Similar results were obtained in MNNG-treated cells (see Figure 4A–C). The WT cells showed a similar overcompensation in NAD recovery at 2.5 μM MNNG, albeit at the earlier time of 8 h, and the TZR cells again showed a delayed recovery (e.g 5 μM MNNG, see 4B) or, in the case of 10 μM MNNG (4C), a complete inability to recover NAD levels at all.

### DNA strand break levels

A 1-h TM treatment resulted in a concentration-dependent increase in DNA strand break levels in both cell lines (Figure 5A). There was a small increase in the net levels of DNA strand breaks per TM dose in the TZR cell line compared with the WT, but this was not significant at concentrations > 500 μM. Co-incubation of a fixed concentration of TM (150 μM) with increasing concentrations of NU1025 resulted in increasing RE values for both cell lines (Figure 5B), indicating that NU1025 retarded DNA strand break rejoining. The RE values for the TZR cells were initially higher than the WT in the absence of NU1025, confirming the results in Figure 5A, and increased at a faster rate with increasing concentration of NU1025 compared with the WT cells. The slopes were significantly different, with values of 0.218 ± 0.018 ($r^2$ = 0.911) (TZR) and 0.166 ± 0.013 ($r^2$ = 0.93) (WT). NU1025 treatment itself (300 μM for 24 h) did not cause DNA strand breakage (results not shown).

### Regulation of NMNAT activity

We reasoned that, because of the dramatic increase in the catabolism of NAD in response to DNA damage, increases in NAD biosynthetic activity, mediated for example by increases in NMNAT levels (e.g. by transcriptional induction) or activity [e.g. post-translational modification by phosphorylation or poly(ADP–ribosylation)], could be important in the regulation of NAD metabolism. This was suggested by the observation that, after low doses of MNNG or TM, there was a time-dependent depletion of NAD followed by a recovery to greater than control levels (see Figures 3B and 4B).

Levels of NMNAT activity in cell extracts were examined from cells treated with MNNG in the presence or absence of 100 μM NU1025 (see Table 2). In cells treated with high concentrations of MNNG (10 μM) for up to 4 h, although there was a slight increase in NMNAT activity compared with control (untreated) cells at 1 h followed by a decrease at later times, there was no significant trend.

### DISCUSSION

Cells can lose ≥ 95% of NMNAT function with only a consequent approximate 50% depletion in NAD levels, indicating that during normal, unstressed growth the reserve capacities of this enzyme and its product NAD are well in excess of requirements. NAD performs pleiotropic and essential cellular functions, both as a

© Cancer Research Campaign 1997

co-factor in oxidation–reduction reactions and as a substrate for poly- and mono-ADP–ribosylation reactions. However, it is well established that the majority of NAD (>90%) is confined to the nucleus, and its turnover is accounted for by poly(ADP–ribose) synthesis (Rechsteiner et al, 1976).

Because NMNAT is an enzyme that must respond to sudden increases in demands on its activity following DNA damage, we reasoned that its activity might be modulated [e.g. by post-translational modification by poly(ADP–ribosylation)] in response to DNA damage. Emanuelli et al (1992) observed that high concentrations of ADP–ribose inhibited NMMAT. However, no evidence for this was found after MNNG treatment, in either the presence or absence of NU1025 (see Table 2).

Compared with WT, the TZR cell line was more sensitive to the growth-inhibitory effects of both TM and MNNG, particularly above concentrations that caused a ≥ 50% NAD depletion, and which took up to 24 h to recover (e.g. 4 or 5 μM MNNG). This suggests that the causative cytotoxic mechanism may involve an irreversible NAD depletion (see also discussion below).

The TZR cell line is approximately two- to three-fold more sensitive to the chemopotentiating effects of NU1025 when used in conjunction with either TM or MNNG. This differential sensitivity can be explained most plausibly by a more effective competitive inhibition of PADPRP by NU1025 in intact cells because of reduced levels of endogenous NAD (approximately 60% of WT) in the TZR cell line competing for binding to the active site of the enzyme. Note that this markedly enhanced sensitivity of the TZR cell line cannot be explained by an alteration in either the activity of PADPRP (which was only approximately 20% lower than WT), although this could be a contributing factor, or its sensitivity to the inhibitor (e.g. by mutation), as the $IC_{50}$ value of NU1025 for inhibition of PADPRP in the in vitro enzyme assay was not significantly different in the two cell lines (see Table 1).

Consistent with the above interpretations is the observation that an analogous increase in the sensitivity to NU1025, in this case relating to its ability to increase net DNA strand break levels in TM-treated cells, was observed in the TZR cell line compared with WT. The slight increase, compared with WT, in DNA strand break levels in TZR cells treated with TM alone suggests that PADPRP function may be compromised by the low substrate levels in the TZR cells.

In marked contrast, the lack of a differential sensitivity between the two cell lines to NU1025 when used by itself indicates that these cytostatic effects [obtained only at much higher concentrations (approximately tenfold) than required for chemopotentiation] are not due to inhibition of PADPRP. Presumably a secondary metabolic effect of this compound becomes manifest at millimolar concentrations.

Although NAD levels were approximately 50% lower in TZR cells than WT, both cell lines demonstrated proportionate dose- and time-dependent depletions in NAD levels after treatment with TM or MNNG. However, because the TZR cell line had lower control levels of NAD to start with, the extent of the depletion was much more severe. For example, 5 μM MNNG reduced NAD levels from approximately 6070 to approximately 4512 pmol $mg^{-1}$ protein by 6 h in WT cells, and from approximately 3678 to approximately 1441 pmol $mg^{-1}$ protein in TZR cells. Thus, TZR cells attained a nadir of 24% of WT NAD levels compared with 74% for WT cells. Furthermore, WT cells recovered normal NAD levels by 8 h, but TZR cells had still not recovered normal levels by 16 h. At higher doses of MNNG (10 μM), TZR cells were completely unable to recover NAD levels from a nadir of 280 pmol $mg^{-1}$ protein over a 24-h time period, whereas the WT cells did. These results are consistent with the low levels of NMNAT in the TZR cell line becoming rate limiting for NAD synthesis under conditions of high levels of DNA damage. A similar pattern of results, although not so extreme, was obtained with TM-treated cells. These data suggest that an irreversible NAD depletion, leading to cell death (Berger, 1985), may contribute to the enhanced cytotoxicity of MNNG and TM alone to TZR cells, observed particularly at higher doses of the drugs.

Tumour tissues of a variety of types have been shown to have lower NAD levels than homologous normal tissue (Jedeiken and Weinhouse, 1955; Glock and Mclean, 1957). Hypoxic tumour cells have increased cellular NADH/NAD ratios, which can reduce available NAD levels at least three-fold (Wilson et al, 1977). In addition, NADH will act as a potent inhibitor of PADPRP (Ueda et al, 1982) with a $K_i$ value of 5 μM (in the same range as 3-aminobenzamide, $K_i$ 1.8 μM, Purnell and Whish, 1980). Studies have indicated that NMNAT activity can vary widely (up to ten-fold) in different cell lines (Ahluwalia et al, 1984), and early, carefully performed studies in mice showed a large reduction in NMNAT activity in a number of tumour types compared with normal tissue (Branster and Morton, 1956). Taken together, these lines of evidence suggest that tumours are likely to have low NAD levels compared with normal tissue.

Modulation of PADPRP activity as a therapeutic strategy is a two-edged sword. On the one hand, extreme PADPRP activation promotes irreversible NAD depletion; on the other hand, PADPRP inhibition inhibits repair while maintaining NAD and ATP pools. The effects that prevail as determinants of cytotoxicity depend on a number of factors. Examples of such factors include initial NAD levels, which are rapidly attenuated by nutritional deprivation of niacin (Fu et al, 1989) and may vary widely in different tissue and tumour types (see above). Furthermore, the dependence on PADPRP function and normal NAD levels for the nucleosomal DNA fragmentation and ensuing apoptosis (Wright et al, 1996; Yoon et al, 1996), as well as the specific proteolytic cleavage of PADPRP (Kaufmann et al, 1993), indicate an important role for PADPRP in programmed cell death caused by chemotherapeutic agents.

We have demonstrated that cellular PADPRP function is readily attenuated by modest changes in NAD concentration, such as probably occur in solid tumours, or by nutritional deprivation of niacin. This reduces the cellular capacity to survive and repair DNA damage and, importantly, sensitizes cells to the chemopotentiating effects of PADPRP inhibitors.

These data provide a rationale for targeting PADPRP function or NAD synthetic enzymes as potentially selective chemotherapeutic strategies for solid tumours. Furthermore, the development of collateral sensitivity to TM and PADPRP inhibitors in TZ-resistant tumours would present the opportunity for 'Yin Yang' chemotherapy, as postulated by Cheng et al (1983).

### ACKNOWLEDGEMENT

This work was supported by a grant from the North of England Cancer Research Campaign.

### ABBREVIATIONS

$IC_{50}$, concentration that reduces growth/activity by 50%; MNNG, 1-methyl-3-nitro-1-nitroso-guanidine; NMNAT, nicotinamide

 © Cancer Research Campaign 1997

mononucleotide adenylyltransferase; PADPRP, poly(ADP–ribose) polymerase; RE, relative elution; TM, temozolomide; TZ, tiazofurin; TZR, tiazofurin resistant; WT, wild type.

### REFERENCES

Ahluwalia GS, Jayaram HN, Plowman JP, Cooney DA and Johns DG (1984) Studies on the mechanism of action of 2-β-D-ribofuranosylthiazole-4-carboxamide –V. Factors governing the response of murine tumours to tiazofurin. *Biochem Pharmacol* **33**: 1195–1203

Althaus FR, Hofferer L, Kleczkowska HE, Malanga M, Naegeli H, Panzeter P and Realini C (1993) Histone shuttle driven by the automodification of poly(ADP-ribose) polymerase. *Environ Mol Mutagen* **22**: 278–282

Balducci E, Emanuelli M, Magni G, Rafaelli N, Ruggieri S, Vita A and Natalini P (1992) Nuclear matrix associated NMN adenylyltransferase activity in human placenta. *Biochem Biophys Res Commun* **189**: 1275–1279

Berger NA (1985) Symposium: cellular response to DNA damage: the role of poly(ADP-ribose). *Radiat Res* **101**: 4–15

Bernofsky C and Swan M (1973) An improved cycling assay for nicotinamide adenine dinucleotide. *Anal Biochem* **53**: 452–458

Boulton S, Pemberton LC, Porteous JK, Curtin NJ, Griffin RJ, Golding, BT and Durkacz BW (1995) Potentiation of temozolomide-induced cytotoxicity: a comparative study of the biological effects of poly(ADP-ribose) polymerase inhibitors. *Br J Cancer* **72**: 849–856

Bradford MM (1976) A rapid and sensitive method for the determination of microgram quantities of protein utilising the principle of protein-dye binding. *Anal Biochem* **72**: 248–254

Branster MV and Morton RK (1956) Comparative rates of synthesis of diphosphopyridine nucleotide by normal and tumour tissue from mouse mammary gland: studies with isolated nuclei. *Biochem J* **63**: 640–646

Cheng Y-C and Brockman WR (1983) Mechanisms of drug resistance and collateral sensitivities: bases for development of chemotherapeutic agents. In *Development of Target-oriented Anticancer Drugs*, Cheng Y-C, Goz B and Minkoff M (eds), pp. 107–117. Raven Press: New York

Cohen A and Barankiewicz J (1987) Metabolic consequences of DNA damage: alteration in purine metabolism following poly(ADP ribosyl)ation in human T-lymphoblasts. *Arch Biochem Biophys* **258**: 498–503

Cooney DA, Jayaram HN, Gebeyehu G, Betts CR, Keeley JA, Marquez VE and Johns DG (1982) The conversion of 2-β-D-ribofuranosylthiazole-4-carboxamide to an analogue of NAD with potent IMP dehydrogenase inhibitory properties. *Biochem Pharmacol* **31**: 2133–2136

de Murcia G and Ménissier-de Murcia JM (1994) Poly(ADP-ribose) polymerase: a molecular nick-sensor. *Trends Biochem Sci* **19**: 172–176

Durkacz BW, Omidiji O, Gray DA and Shall S (1980) (ADP-ribose)$_n$ participates in DNA excision repair. *Nature* **283**: 593–596

Emanuelli M, Natalini P, Raffaelli N, Ruggieri S, Vita A and Magni G (1992) NAD biosynthesis in human placenta: purification and characterisation of homogeneous NMN adenylyltransferase. *Arch Biochem Biophys* **296**: 29–34

Fornace AJ Jr and Little JB (1977) DNA crosslinking induced by X-rays and chemical agents. *Biochim Biophys Acta* **477**: 343–355

Fu CS, Swendseid ME, Jacob RA and McKee RW (1989) Biochemical markers for niacin status in young men: levels of erythrocyte niacin coenzymes and plasma tryptophan. *J Nutr* **119**: 1949–1955

Glock GE and McLean P (1957) Levels of oxidised and reduced diphosphopyridine nucleotide and triphosphopyridine nucleotide in tumours. *Biochem J* **65**: 413–416

Griffin RJ, Pemberton LC, Rhodes D, Bleasdale C, Bowman K, Calvert AH, Curtin NJ, Durkacz BW, Newell DR, Porteous JK and Golding BT (1995) Novel potent inhibitors of the DNA repair enzyme poly(ADP-ribose) polymerase (PARP). *Anticancer Drug Design* **10**: 507–514

Grube K, Küpper JH and Bürkle A (1991) Direct stimulation of poly(ADP-ribose) polymerase in permeabilised cells by double-stranded DNA oligomers. *Anal Biochem* **193**: 236–239

Halldorsson H, Gray DA and Shall S (1978) Poly(ADP-ribose) polymerase activity in nucleotide permeable cells. *Febs Letts* **85**: 349–352

Heller B, Wang ZQ, Wagner EF, Radons J, Bürkle A, Fehsel K, Burkart V and Kolb H (1995) Inactivation of the poly(ADP-ribose) polymerase gene affects oxygen radical and nitric oxide toxicity in islet cells. *J Biol Chem* **270**: 11176–11180

Jacobson EJ, Nunbhakdi-Craig V, Smith DG, Chen HY, Wasson BL and Jacobson MK (1992) ADP-ribose polymer metabolism: implications for human nutrition. In *ADP–Ribosylation Reactions*, Poirier GG and Moreau P (eds), pp. 153–162. Springer: New York

Jayaram HN, Cooney DA, Glazer RI, Dion RL and Johns DG (1982) Mechanism of resistance to the oncolytic C-nucleoside, 2-β-D-ribofuranosylthiazole-4-carboxamide (NSC286193). *Biochem Pharmacol* **31**: 2557–2560

Jayaram HN, Zhen W and Gharebaghi K (1993) Biochemical consequences of resistance to tiazofurin in human myelogenous leukemic cells. *Cancer Res* **53**: 2344–2348

Jedeiken LA and Weinhouse S (1955) Metabolism of neoplastic tissue VI. Assay of oxidised and reduced diphosphopyridine nucleotide in normal and neoplastic tissues. *J Biol Chem* **213**: 271–280

Kaufmann SH, Brunet G, Talbot B, Lamarr D, Dumas C, Shaper JH and Poirier G (1991) Association of poly(ADP-ribose) polymerase with the nuclear matrix: the role of intermolecular disulfide bond formation, RNA retention and cell type. *Exp Cell Res* **192**: 524–535

Kaufmann SH, Desnoyers S, Ottaviano Y, Davidson NE, and Poirier GG (1993) Specific proteolytic cleavage of poly(ADP-ribose) polymerase: an early marker of chemotherapy-induced apoptosis. *Cancer Res* **53**: 3976–3985

Kohn KW, Ewig RAG, Erickson LC and Zwelling LA (1981) Measurement of strand breaks and crosslinks by alkaline elution. In *DNA Repair: A Laboratory Manual of Research Procedures*, Friedberg EC and Hanawalt PC (eds), vol. 1, part B, pp. 379–401. Marcel Dekker: New York

Lautier D, Lagueux J, Thibodeau J, Ménard L and Poirier GG (1993) Molecular and biochemical features of poly(ADP-ribose) metabolism. *Mol Cell Biochem* **122**: 171–193

Lindahl T, Satoh MS, Poirier GG and Klungland A (1995) Post-translational modification of poly(ADP-ribose) polymerase induced by DNA strand breaks. *Trends Biochem Sci* **20**: 405–411

Molinette M, Vermeulen W, Bürkle A, Ménissier-de Murcia J, Küpper JH, Hoeijmakers JH and De Murcia G (1993) Overproduction of the poly(ADP-ribose) polymerase DNA-binding domain blocks alkylation-induced DNA repair synthesis in mammalian cells. *EMBO J* **12**: 2109–2117

Newlands ES, Blackledge GRP, Slack JA, Rustin GJS, Smith DB, Stuart NSA, Quarterman CP, Hoffman R, Stevens MFG, Brampton MH and Gibson AC (1992) Phase I trial of temozolomide (CCRG 81045: M & B 39831: NSC 362856). *Br J Cancer* **65**: 287–291

Purnell MR and Whish WJD (1980) Novel inhibitors of poly(ADP-ribose) synthase. *Anal Biochem* **27**: 212–217

Radons J, Heller B, Bürkle A, Hartmann B, Rodriguez ML, Kroncke KD, Burkhart V and Kolb H (1994) Nitric oxide toxicity in islet cells involves poly(ADP-ribose) polymerase activation and concomitant NAD depletion. *Biochem Biophys Res Commun* **199**: 1270–1277

Rechsteiner M, Hillyard D and Olivera BM (1976) Magnitude and significance of NAD turnover in human cell line D98/AH2. *Nature* **259**: 695–696

Stevens MFG, Hickman JA, Langdon SP, Chubb D, Vickers L, Stone R, Baig G, Goddard C, Gibson NW, Slack JA, Newton C, Lunt E, Fizames C and Lavelle F (1987) Antitumor activity and pharmacokinetics in mice of 8-carbamoyl-3-methyl-imidazo[5,1-*d*]-1,2,3,5-tetrazin-4(3*H*)-one (CCRG81045; M & B 39831), a novel drug with potential as an alternative to decarbazine. *Cancer Res* **47**: 5846–5852

Suto MJ, Turner WR, Arundel-Suto CM, Werbel LM and Sebolt-Leopold JS (1991) Dihydroisoquinolinones: the design and synthesis of a new series of potent inhibitors of poly(ADP-ribose) polymerase. *Anticancer Drug Design* **7**: 101–107

Ueda K, Kawaichi M and Hayaishi O (1982) Poly(ADP-ribose) synthetase. In *ADP-Ribosylation Reactions*, Hayaishi O and Ueda K (eds), pp. 117–155. Academic Press: NY

Wang Z-Q, Auer B, Stingl L, Berghammer H, Haidacher D, Schweiger M and Wagner EI (1995) Mice lacking ADPRT and poly(ADP-ribosyl)ation develop normally but are susceptible to skin disease. *Genes Dev* **9**: 509–520

Wilson DF, Erecinska M, Brown C and Silver IA (1977) Effect of of oxygen tension on cellular energetics. *Am J Physiol* **233**: C135–C140

Wright SC, Wei QS, Kinder DH and Larrick JW (1996) Biochemical pathways of apoptosis: nicotinamide adenine dinucleotide-deficient cells are resistant to tumour necrosis factor or ultraviolet light activation of the 24-kD apoptotic protease and DNA fragmentation. *J Exp Med* **183**: 463–471

Yoon YS, Kim JW, Kang KW, Kim YS, Choi KH and Joe CO (1996) Poly(ADP-ribosyl)ation of histone H1 correlates with internucleosomal DNA fragmentation during apoptosis. *J Biol Chem* **271**: 9129–9134

Zhang J, Dawson VL, Dawson TM and Snyder SH (1994) Nitric oxide activation of poly(ADP-ribose) synthetase in neurotoxicity. *Science* **263**: 687–689

© Cancer Research Campaign 1997